# First Case of Mature Teratoma and Yolk Sac Testis Tumor Associated to Inherited MEN-1 Syndrome

**DOI:** 10.3389/fendo.2019.00365

**Published:** 2019-06-12

**Authors:** Sabrina Chiloiro, Ettore Domenico Capoluongo, Giovanni Schinzari, Paola Concolino, Ernesto Rossi, Maurizio Martini, Alessandra Cocomazzi, Giuseppe Grande, Domenico Milardi, Brigida Anna Maiorano, Antonella Giampietro, Guido Rindi, Alfredo Pontecorvi, Laura De Marinis, Antonio Bianchi

**Affiliations:** ^1^UOC di Endocrinologia e Diabetologia, Fondazione Policlinico Universitario A. Gemelli, IRCCS, ENETS Center of Excellence, Istituto di Patologia Speciale Medica, Università Cattolica del Sacro Cuore, Rome, Italy; ^2^OUC di Oncologia Medica, Fondazione Policlinico Universitario A. Gemelli, IRCCS, ENETS Center of Excellence, Università Cattolica del Sacro Cuore, Rome, Italy; ^3^Area di Diagnostica di Laboratorio Fondazione Policlinico Universitario A. Gemelli, IRCCS, ENETS Center of Excellence, Università Cattolica del Sacro Cuore, Rome, Italy; ^4^OUC di Anatomia Patologica, Fondazione Policlinico Universitario A. Gemelli, IRCCS, ENETS Center of Excellence, Università Cattolica del Sacro Cuore, Rome, Italy

**Keywords:** menin, SNP, neuroendocrine tumor, insulinoma, hyperparathyroidism

## Abstract

**Introduction:** Multiple endocrine neoplasia type 1 (MEN1) is an autosomal dominantly inherited endocrine tumor syndrome characterized by the development of cancer in various endocrine organs, particularly in the pituitary, parathyroid and pancreas. Moreover, in some cases, also non-endocrine tumors can be diagnosed, developing atypical phenotypes.

**Case report:** We report herein the clinical history of a patient affected by MEN-1 syndrome who developed atypical features for this disease. The patient's clinical history started in August 2015 when he was referred, at the age of 23 years, to the Emergency Department of our Hospital for the occurrence of progressive asthenia, weakness, tremors and syncope. The biochemical test documented hyper-calcemia and severe hypoglycemia. The patient was referred to our Neuroendocrine Tumor and Pituitary Unit and he was diagnosed with pancreatic insulinoma, hypercalcemic hyperparathyroidism, and a prolactin secreting pituitary adenoma. The MEN-1 syndrome was suspected and genetic tests for mutation of *menin* resulted positive for the pathogenic variant c1548dupG. In January 2016, the patient was diagnosed with intratubular germ cell neoplasia, consisting of a mature teratoma and yolk sac tumor and he underwent a right orchiectomy.

**Conclusion:** This is the first case report showing the clear association of MEN-1 syndrome with yolk sac tumors and teratomas, as in our case, the c1548dupG represents a pathogenic variant rather than a SNP. This case suggests the opportunity of an accurate evaluation of the testis particularly in young MEN-1 affected patients and that a prompt screening for neoplastic disease should involve all the endocrine glands.

## Introduction

Multiple endocrine neoplasia type 1 (MEN1) is an autosomal dominantly inherited endocrine tumor syndrome characterized by tumor development in various endocrine organs (1, 2). MEN syndromes are infrequent inherited disorders in which more than one endocrine gland develops noncancerous (benign) or cancerous (malignant) tumors or grows excessively without forming tumors. MEN1 disease is a consequence of the *MEN1* gene mutation (3–5). The *MEN1* gene synthetizes the protein menin, that acts as a tumor suppressor, as confirmed by microsatellite analysis conducted on cancerous tissues of MEN1 patients (6, 7). The protein menin inhibits the cell proliferation through the interaction with histone-modifying enzymes, with transforming growth factor β1 (TGF-β) signaling and Wnt/β-catenin pathways and with several transcription factors (such as nuclear factor κB (NF-κB), peroxisome proliferator-activated receptors (PPARγ), and vitamin D receptor (VDR) (8). Moreover, menin can act by destroying pro-proliferative factors such as insulin-like growth factors I and II (IGF-I and IGF-II) and parathyroid hormone-related protein (PTHrP) (8). MEN-1 syndrome can present as a familial form (more common) or sporadic form. Specific gene mutations can be identified in 70–95% of cases (3–9). The most commonly diagnosed tumors in MEN-1 syndrome involve the parathyroid glands in around 95% of cases, endocrine pancreatic-gastroenteric tract in around 40% of cases and the anterior pituitary gland, in around 30% of cases (10, 11). The first presentation of MEN1, in up to 85% of patients, is a parathyroid tumor; in other cases, the first manifestation may be prolactinoma or an insulinoma (12). Other tumors can occur in MEN-1 syndrome such as adrenocortical and thyroid tumors, meningiomas, angiofibromas, collagenomas, lipomas and gastric, thymic, and bronchial carcinoids (13–19). Notably, MEN-1 syndrome can show a very variable phenotype (9). We report herein the clinical history of a patient affected by MEN-1 syndrome who developed atypical features for this disease. This feature is peculiar as it has never been described in literature. A written informed consent was obtained from the patient for the publication of this case report and any potentially-identifying images/information.

## Case Report

The patient's clinical history started at the age of 15 years, when he was diagnosed for minor epilepsy. The patient's actual clinical history started in August 2015 when he was referred, at the age of 23 years, to the Emergency Department of our Hospital for the occurrence of progressive asthenia, weakness, tremors and syncope. The biochemical test documented hypercalcemia and severe hypoglycemia. The glycemic value was 27 mg/dL. The patient was treated with a glucose infusion with symptoms reduction. In September 2015, the patient was admitted to our Neuroendocrine Tumor and Pituitary Unit, to perform a 72 h fasting test for a possible insulinoma. After 7 h fasting, the patient was symptomatic for hypoglycemia. The glycemic plasma value resulted as 20 mg/dL, insulin as 18.6 microIU/mL, C-peptide as 1.7 ng/mL. Again symptoms diminished following the glucose infusion. Additionally, blood tests documented a primary hyperparathyroidism with hypercalcemia (Calcium: 11.7 mg/dL, PTH: 134.5 pg/mL) and hyperprolactinemia (PRL: 220 ng/mL).

**Figure 1:**
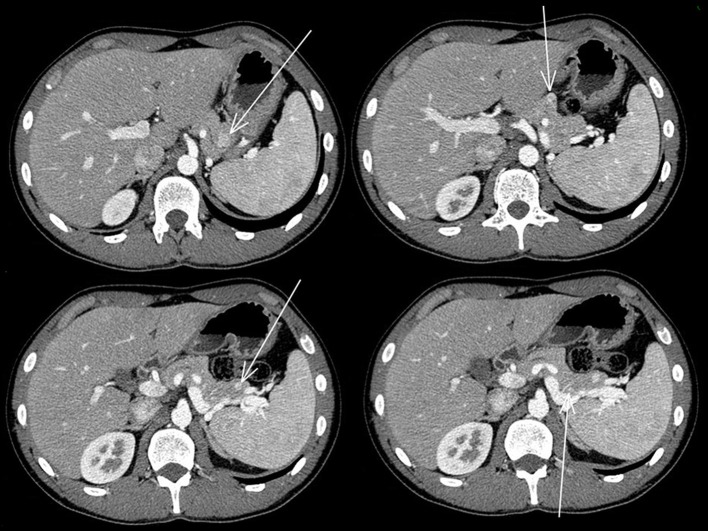
Abdominal contrasted TC scan showed the four pancreatic neuroendocrine tumors that are indicated with the arrows.

**Figure 2:**
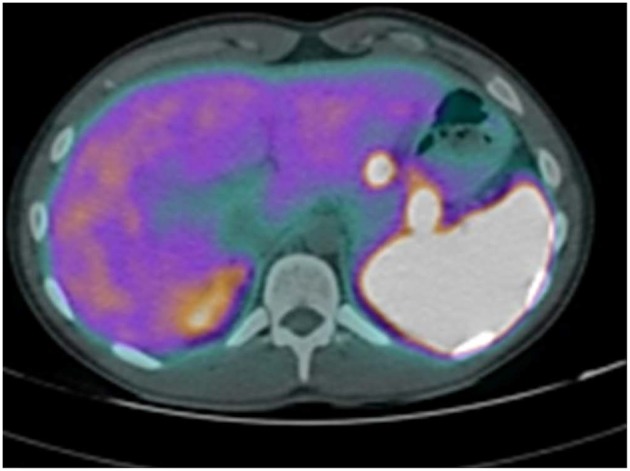
Gallium-68 labeled somatostatin receptor PET-CT showed uptake in 3 Gallium-68 labeled somatostatin receptor PET-CT showed an uptake in 3 nodules in the pancreas.

The abdominal contrast computerized tomography (CT) documented the presence of four hyper-vascular focal lesions, of <1 centimeter and localized at the pancreatic body and tail, which were suggestive for neuroendocrine tumors (NET) (Figure 1). A Gallium-68 labeled somatostatin receptor PET-CT an showed uptake in 3 nodules in the pancreas (Figure 2). Cytological findings of the endoscopic ultrasound-guided fine needle aspiration of the larger pancreatic tumor was consistent with a G2 neuroendocrine tumor, with positive immunohistochemistry for chromogranin A, synaptophysin, CDX2 and a Ki67 proliferation index of 4%. Based on the patient's clinical history, immunohistochemistry was performed for insulin and resulted positive in tumor cells. The patient underwent a thyroid and parathyroid ultrasound which resulted negative for both thyroid nodules and hyperplastic parathyroid. The parathyroid scintigraphy however showed two hyper-functioning parathyroid glands. A pituitary contrasted magnetic resonance evidenced the presence of a small pituitary adenoma with a maximum size of 8 millimeters. Consequently, the patient initiated a prophylactic treatment with diazoxide (at the starting dosage of 25 mg/daily with a subsequent dose titration up to 75 mg/daily) to prevent a potential hypoglycemia crisis, with long acting somatostatin analogs (SSA: Lanreotide Autogel 120 mg/monthly) for the pancreatic NET and with a dopamine agonist (cabergoline 0.5 mg half table twice a week) for the micro-prolactinoma.

In the family history, the patient's sister underwent successful neurosurgery to remove a pituitary prolactinoma at the age of 18 years.

According to the patient's clinical assessment and family history, a MEN-1 syndrome was suspected. Genetic testing for the mutation of *menin* resulted positive for the pathogenic variant c1548dupG, in heterozygosis.

**Figure 3:**
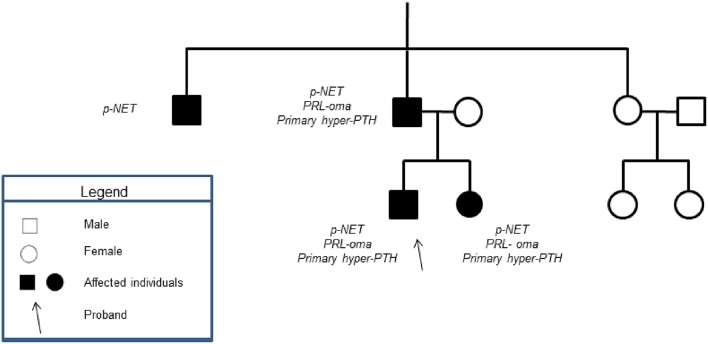
Showed the patient's family tree. None of the patient's male relatives had history of testicular mass.

All of the patients' first-degree relatives were tested for MEN-1 syndrome, after signing informed consents. Figure 3 shows the index pedigree.

According to the multidisciplinary decision of the Neuroendocrine tumor (NET) board, the patient first received a total parathyroidectomy before the scheduled sub-total pancreatectomy treatment.

In November 2015, a total parathyroidectomy and a thymectomy were performed. The dosage of intra-operatory serum PTH showed a progressive reduction, from the initial value of 191.3 pg/mL to the final value of 20 pg/mL. The patient was treated with calcitriol without any occurrence of hypocalcemia. The histological examination documented a diffuse hyperplasia of all four removed parathyroid glands in the absence of thymic neoplasia/hyperplasia and only initial adipose thymic involution. One month after surgery, serum PTH concentration was of 5 pg/mL (range 14–72).

**Figure 4:**
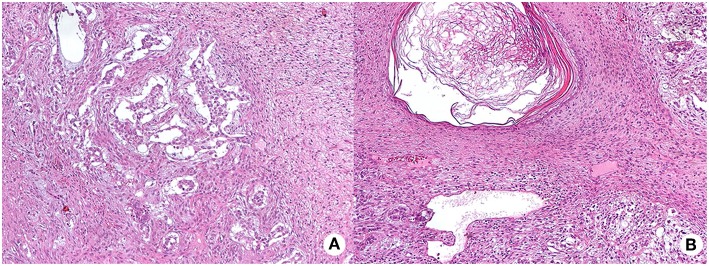
Hematoxylin and eosin (HE) staining of testis intra-tubular germ cell neoplasia (200X magnification) composed by the yolk sac tumor, with mainly anastomosing channels that focally expand to form variably sized cysts lined by primitive tumor cells with varying amounts of clear, glycogenated cytoplasm (mycrocistic or reticular pattern, panel **A**) and of the mature teratoma, with different type of mature tissue such as squamous epithelium with keratinization (panel **B** shows a dermoid cyst).

In January 2016, the patient was referred with right testicular swelling. The alpha-fetoprotein serum level was 43 ng/mL (<9). A testis ultrasound documented a hypoechoic and hyper-vascularized nodule. The patient underwent a testicular nodule resection. The histological examination showed an intratubular germ cell neoplasia (IGCNU), consisting of a mature teratoma and yolk sac tumor, with signs of pre-invasive lesion, such as presence of peri-neoplastic, placental alkaline phosphatase (PLAP) and CD117 positive seminiferous tubules, with basal nuclei and abundant clear cytoplasm (Figure 4). According to the presence of germ cell neoplasia, in March 2016, a right orchiectomy was conducted, following germ cell cryopreservation. No neoplastic cells were detected at the histological examination of the resected testis. The post-surgery total body CT and 18F-FDG PET/CT were negative for metastasis 6 months later.

In February 2017, the patient underwent a sub-total pancreatectomy. Treatment with diazoxide was withdrawn.

The histological examination proved the presence of four pancreatic NETs: two of the pancreas body and the other two at the pancreas tail. All the lesions showed a positive immunohistochemistry for chromogranin A and synaptophysin and one only was positive for insulin. The higher mitotic index was 7 per 10 high-power fields with the Ki67 proliferation index ranging from 4 to 8%. These lesions were diagnosed as three non-functioning G2 NET and one insulinoma G2 NET. No other histological alterations were identified in the endocrine and in the exocrine residual pancreas.

At present the patient is in good clinical condition, in the absence of disease recurrence or adverse event such as diabetes mellitus or episodes of hypocalcemia or hypoglycemia. He is still on treatment with SSA and DA, with normal prolactin values. Hormonal replacement therapy with testosterone analogs was not prescribed, given the absence of referred symptoms and according to the laboratory assessment.

Blood test, abdominal CT, Gallium-68 labeled somatostatin receptor PET-CT and pituitary MR are periodically scheduled at our Neuroendocrine Tumor and Pituitary Unit.

## Discussion

To our knowledge, this is the first MEN1 patient who also developed an intra-tubular germ cell neoplasia of the testis. It is well-known that endocrine glands are very sensitive to the development of noncancerous or cancerous lesions. In a recent study by Wautot et al. (20) the menin expression (detected as a 68 KDalton protein) was demonstrated in the brain cortex, the kidney, the pituitary, the testes, the thymus and in the thyroid, providing a rationale for the high risk of neoplasia development at these sites in *MEN1* patients.

We can speculate that the germline c1548dupG pathogenic variant could also play a role in the onset of this peculiar tumor of the testis. In this regard, we also tried to evaluate the status of *MEN1* copy number (CNV), however and unfortunately, we could not assess CNV since our method is set for germline blood-derived fresh DNA rather than on somatic formalin-fixed paraffin-embedded (FFPE) DNA (data not shown). As reported, MEN1 gene encodes for menin that act as tumor suppressor, as confirmed by microsatellite analysis conducted on cancerous tissues of *MEN1* patients. Therefore, although in the absence of a direct evidence, we cannot exclude the relationship between *MEN1*-mutations and testis tumor development. In addition, the c.1548dupG (p.Lys517Glufs; rs761695866) is very well-established as a pathogenic variant and reported as very rare within the population (Varsome Database). The absence of *MEN1* mutations reported for yolk sac and mature teratoma testis tumor (the tumor described in this report), within the ATLAS genome and COSMIC databases may well be due to the rarity of this tumor histotype. The testis tumor affecting our patient was classified as a non-germinomatous germ cell tumor (NGGCTs) (21) or as a type II testicular germ cell tumor (22). This group of testis neoplasia typically occurs in the third and fourth decade of life and includes seminoma, embryonal carcinoma, teratoma, yolk sac tumor, choriocarcinoma, and mixed germ cell tumors (22). All type II testicular germ cell tumors develop from a pre-invasive lesion called intratubular germ cell neoplasia unclassified (IGCNU), defined as malignant germ cells confined to the seminiferous tubules, which usually lack normal spermatogenesis (22).

Similarly, teratomas derive from pluripotent cells (23) and can be differentiated in mature and immature, according to the differentiation grade of tissue within the tumors. Fully differentiated neuroectodermal, mesodermal and endodermal elements are detected in mature teratomas. Instead, embryonic elements deriving from any or all of the three germinal cell layers are typically detected in immature teratomas (23). Teratomas are commonly located in gonads, anterior mediastinum, retroperitoneum, and sacrococcygeal region but can also involve atypical organs, such as the pituitary gland (24).

Similar to other neoplasia in the testis, several factors were suggested as being involved into the onco-genesis, such as genetic disorders and a history of cryptorchidism or testis dysgenesis. Genetic studies have suggested an association between testis oncogenesis and mutations of several genes. In particular, since 2009 there are new genetic insights starting from two testicular germ cell tumors (TGCT)- genome wide association studies (GWAS), followed by several additional TGCT-GWAS (25). In these studies some SNPs with significant associations were identified in or near the genes KITLG (KIT ligand), SPRY4 (sprouty 4: sprout-related, EVH1 domain containing 2), BAK1 (BCL2-antagonist/killer 1), DMRT1 (doublesex and mab-3-related transcription factor 1), TERT (telomerase reverse transcriptase), ATF7IP (activating transcription factor 7 interacting protein), HPGDS (hematopoietic prostaglandin D synthase), MAD1L1 (mitotic arrest deficient-like 1), RFWD3 (ring finger WD domain 3), TEX14 (testis expressed 14), and PPM1E (protein phosphatase, Mg2+/Mn2+ dependent, 1E) (25).

We underline the fact that the Elzinga-Tinke et al. review paper does not associate *MEN1* gene pathogenic variants to the etiopathogenesis of TGCT, so this is the first case report showing a clear association with yolk sac tumors and with teratomas. Furthermore, all the above mentioned GWAS studies identified only SNPs within the called genes. In our case, however, the c1548dupG represents a pathogenic variant rather than a SNP variant. This data can further support the association between our peculiar phenotype with the genotype.

This case report confirms that the early diagnosis of MEN-1 syndrome, along with appropriate screening and prompt therapeutic management of MEN-1 related neoplasia, can improve prognosis, particularly in cases of pancreatic NET, as shown in our previous experience (26, 27). In fact, in most cases, MEN-1 related tumors are diagnosed for local mass effects or for symptoms due to the overproduction of hormones (12). Although MEN-1 related tumors are usually benign, an aggressive behavior, with high risk of malignancy, as for carcinoid tumors and gastrinomas can occur (28). Consequently, in individuals with two or more MEN1-related tumors and in first-degree family members, a genetic test for MEN-1 syndrome should be offered (29). In addition, patients with the genetic diagnosis of MEN-1 syndrome should also be offered an appropriate screening and follow-up for all MEN-1 related tumors.

According to our experience and clinical practice, the integration of diagnostic modalities can improve the sensibility of each diagnostic test allowing an earlier and effective diagnosis. In particular, in our case the integration of neck ultrasound and parathyroid scintigraphy allowed the diagnosis of primary hyperparathyroidism. The sensitivity of ultrasonography is 76–87% with a positive predictive value of 93–97% and a diagnostic accuracy of 88% (30). By converse, 99 mTc-sestamibi scintigraphy has a higher sensitivity (90%) and accuracy (97.2%) then ultrasound (30). However, the concordance between scintigraphy and ultrasound is nor reached in all cases (30). On the same line CDX2 immunohistochemistry was conducted on the diagnostic cytological specimens to confirm the digestive source (and namely pancreatic) origin of tumor cells. CDX2 protein expression was reported positive in a percentage of pancreatic neuroendocrine tumors (31, 32). Similarly, PDX1, a transcription factor, was identified in metastatic NET of gastro-intestinal and pancreatic origin (31, 32).

This case suggests the opportunity of an accurate evaluation of testis particularly in affected young MEN-1 patients. Others neoplasia such as thyroid and breast tumors can be detected in patients affected by MEN1 syndrome and required an appropriate screening (33). However, as these neoplasms are common also in the general population and since the role of *MEN1* gene in the thyroid and breast cancers is uncertain, the association of thyroid and breast tumors and *MEN1* is considered incidental (33).

In conclusion, this unique case report suggests that a prompt screening for neoplastic disease should involve all the endocrine glands (not only pituitary, parathyroids, and pancreas), in patients diagnosed for MEN-1 syndrome, in order to have the opportunity and the benefit of an early diagnosis of neoplasia.

## Data Availability

No datasets were generated or analyzed for this study.

## Ethics Statement

This study represents a case report. All the procedures in this case were conducted according to guidelines and according to clinical practice. All procedures performed in studies involving human participants were in accordance with the ethical standards of the Ethics committee of Fondazione Policlinico Gemelli, Rome and with the 1964 Helsinki declaration and its later amendments or comparable ethical standards. A written informed consent was obtained from the patient for the publication of this case report and any potentially-identifying images/information.

## Author Contributions

SC and EC wrote the manuscript. EC, PC, and AC conducted the genetic analysis. GS, ER, and BM conducted the oncological management and follow-up. MM conducted the pathological examination of testis tumor. GR conducted the pathological examination of the neuroendocrine tumors. GG and DM conducted the clinical diagnosis of testis tumor. LD, AB, SC, AG, and AP conducted the endocrinological diagnosis and follow-up. All the authors reviewed and approved the manuscript version.

### Conflict of Interest Statement

The authors declare that the research was conducted in the absence of any commercial or financial relationships that could be construed as a potential conflict of interest.
